# Preoperative exercise tolerance, nutritional-inflammatory markers, and outcomes after TEVAR for type B aortic pathology

**DOI:** 10.3389/fcvm.2026.1844883

**Published:** 2026-06-09

**Authors:** Haiyan Huang, Zhibing Ming, Wei Gu, Yuhong Tan, Jie Dai

**Affiliations:** 1 Department of Vascular Surgery, Nantong First People’s Hospital, Nantong, Jiangsu, China; 2 Department of Vascular Surgery, Nantong First People’s Hospital, Nantong, Jiangsu, China; 3 Department of Neurology, Nantong First People’s Hospital, Nantong, Jiangsu, China

**Keywords:** delayed discharge, exercise tolerance, inflammation, mid-term MAEs, nutrition, thoracic endovascular aortic repair, type b aortic dissection

## Abstract

**Background:**

Thoracic endovascular aortic repair (TEVAR) is the cornerstone treatment for type B aortic dissection (TBAD), but delayed discharge and mid-term major adverse events (MAEs) remain important clinical challenges. This single-center retrospective cohort study aimed to explore the associations of preoperative exercise tolerance, nutritional and inflammatory markers with short-term and mid-term outcomes in patients undergoing thoracic endovascular aortic repair (TEVAR) for type B aortic pathology.

**Methods:**

A retrospective cohort study enrolled 300 consecutive patients who underwent TEVAR for type B aortic pathology (type B acute aortic syndrome, B-AAS) between 2015 and 2020. The study population included three distinct entities: classic TBAD, intramural hematoma (IMH), and penetrating atherosclerotic ulcer (PAU), neither of which are regarded as subtypes of aortic dissection. Preoperative exercise tolerance was assessed by 6-minute walking distance (6MWD) and hand grip strength (HGS), together with geriatric nutritional risk index (GNRI), neutrophil-to-lymphocyte ratio (NLR), and activated partial thromboplastin time (APTT). Primary outcomes were delayed discharge and mid-term MAEs. Delayed discharge was explicitly defined as postoperative hospital stay >10 days, and mid-term major adverse events (MAEs) were recorded during follow-up. Univariate and multivariate regression analyses were performed to identify factors independently associated with outcomes, and interaction analyses were further conducted.

**Results:**

Delayed discharge occurred in 18 (6.0%) patients and mid-term MAEs in 38 (12.7%). Independent predictors of delayed discharge included elevated APTT and NLR, and decreased GNRI, 6MWD, and HGS. The nomogram showed good discrimination (AUC: 0.84). Low 6MWD combined with low GNRI or high NLR synergistically increased the risks of delayed discharge and MAEs (both *P* < 0.001). Restricted cubic spline analyses confirmed linear dose-response relationships.

**Conclusions:**

Preoperative exercise tolerance, nutritional status, and inflammatory biomarkers are independent correlative predictors of short-term and mid-term adverse outcomes after TEVAR. Combined assessment of these simple non-invasive indicators may assist preoperative clinical risk stratification.

## Introduction

Type B aortic dissection (TBAD) is a life-threatening acute aortic syndrome. Thoracic endovascular aortic repair (TEVAR) is the standard first-line treatment for complicated TBAD and high-risk uncomplicated TBAD, and is primarily performed in the acute (<14 days) or subacute phase (14–90 days) after onset given its minimally invasive benefits (1, 2). While TEVAR reduces short-term mortality, delayed discharge (prolonged hospital stay beyond the expected range) remains a common issue, leading to increased healthcare costs and reduced patient satisfaction (3, 4). Additionally, mid-term adverse events (defined as 6 months to 2 years after TEVAR) such as aortic rupture, endoleak, and cardiovascular events still occur in a considerable proportion of patients, affecting long-term survival (5, 6). Therefore, identifying reliable predictors for these outcomes is crucial for optimizing perioperative management and improving prognosis. Moreover, we specifically focused on patients with TBAD rather than those with aortic aneurysm in this study. First, TBAD is an acute, life-threatening aortic syndrome featured by sudden aortic wall injury, severe systemic inflammation and acute catabolic stress, which is distinctly different from chronic aortic aneurysm in pathophysiology (1, 2). As the first-line emergency treatment for TBAD, TEVAR still faces prominent clinical challenges including delayed postoperative recovery and mid-term adverse events (5). Second, even though TBAD tends to occur in younger patients, acute systemic inflammation, nutritional imbalance and cardiopulmonary stress caused by the dissection can still lead to an obvious decline in exercise tolerance. This reduced exercise tolerance reflects impaired physiological reserve and can independently predict postoperative outcomes regardless of age (6). Nutritional status and systemic inflammation are closely associated with postoperative recovery and long-term outcomes in cardiovascular patients (7, 8). The geriatric nutritional risk index (GNRI) and prognostic nutritional index (PNI) have been validated to predict discharge outcomes and mortality in aortic dissection patients (9, 10). Inflammatory indicators like neutrophil-to-lymphocyte ratio (NLR) and platelet-to-lymphocyte ratio (PLR) reflect the body's inflammatory load, and their elevation is linked to poor outcomes after TEVAR (11, 12). Exercise tolerance, as a comprehensive indicator of cardiopulmonary, skeletal muscle, and metabolic function, integrates the effects of nutrition and inflammation (13). Poor nutrition can lead to muscle atrophy, while chronic inflammation impairs cardiopulmonary function, both resulting in reduced exercise tolerance (14). Conversely, low exercise tolerance may indicate compromised physiological reserve, potentially predicting delayed recovery and adverse mid-term events. Existing studies have explored the prognostic value of exercise tolerance in cardiovascular diseases, but few have focused on TBAD patients after TEVAR, and the interactive effects of exercise tolerance with nutritional and inflammatory indicators on delayed discharge and mid-term MAEs remain unclear.

This retrospective cohort study with a median follow-up of 485 days aimed to: (1) evaluate the predictive value of preoperative exercise tolerance for delayed discharge and mid-term MAEs in patients with type B aortic pathology after TEVAR; (2) clarify the interaction between exercise tolerance and nutritional/inflammatory indicators; (3) explore the dose-response relationship between exercise tolerance and the primary outcomes. The findings are expected to provide new insights for preoperative risk stratification and individualized intervention.

## Materials and methods

### Study population

Consecutive patients who underwent TEVAR for type B aortic pathology (B-AAS) at our hospital from January 2015 to December 2020 were enrolled. B-AAS includes three distinct pathological entities: TBAD, intramural hematoma (IMH), and penetrating atherosclerotic ulcer (PAU), the latter two of which are well-defined independent conditions and not classified as subtypes of aortic dissection. Inclusion criteria: (1) Type B aortic pathology (B-AAS: TBAD, IMH, PAU) confirmed by computed tomography angiography (CTA); (2) aged ≥18 years; (3) underwent TEVAR as primary treatment; (4) complete preoperative data (exercise tolerance, nutrition, inflammation indicators); (5) follow-up duration ≥6 months. Exclusion criteria: (1) complicated with type A aortic dissection or preoperative frank aortic rupture; Type A aortic dissection was excluded because it requires emergency open surgery rather than endovascular repair (TEVAR), which has distinctly different pathophysiology, surgical strategy, postoperative recovery patterns and prognosis. This exclusion ensured a homogeneous cohort of TBAD patients treated with TEVAR. (2) malignant tumors, hematological diseases, autoimmune diseases, or severe infections requiring long-term anti-inflammatory/immunosuppressive therapy; (3) previous aortic surgery or TEVAR; (4) neurological or musculoskeletal diseases affecting exercise tolerance assessment; (5) incomplete clinical or follow-up data. A total of 356 patients were initially screened, and 56 were excluded, leaving 300 for final analysis. Indications for TEVAR included complicated B-AAS (impending aortic rupture, malperfusion syndrome, refractory hypertension or pain, rapid aortic expansion) and high-risk uncomplicated TBAD (large aortic diameter, extensive dissection). Disease phases were defined based on the interval from symptom onset to TEVAR: acute (<14 days), subacute (14–90 days), and chronic (>90 days). A total of 217 patients (72.3%) underwent TEVAR in the acute phase (<14 days from onset), and 83 patients (27.7%) in the subacute phase (14–90 days from onset). The study was approved by the Ethics Committee of our Hospital (No. 2024-012) and complied with the Helsinki Declaration. Informed consent was waived due to the retrospective design.

### Outcome definitions

Delayed discharge: Postoperative hospital stay exceeding the 95th percentile of the entire cohort (15). The 95th percentile was calculated based on the postoperative stay of all included patients.

Mid-term MAEs: Follow-up started from the day of TEVAR, with the last follow-up in December 2023. “Mid-term” was defined as 6 months to 2 years after TEVAR, which is the critical period for TEVAR-related adverse events (e.g., endoleak, stent migration, aortic remodeling disorders) and the key time window for evaluating the mid-term efficacy of aortic repair. Mid-term MAEs included new-onset aortic rupture, new-onset retrograde type A aortic dissection, stent graft-induced new entry tear, endoleak, stent graft stenosis/migration/occlusion/infection, aortoesophageal fistula, all-cause mortality, myocardial infarction, stroke, heart failure, and aortic reintervention (5, 6).

### Assessment of exercise tolerance, nutritional and inflammatory indicators

6MWD and HGS were chosen to evaluate exercise tolerance because they objectively reflect global cardiopulmonary function and skeletal muscle strength, which have been validated as prognostic predictors in cardiovascular surgery. GNRI was selected as a validated nutritional assessment tool for aortic disease patients. NLR was included as a representative systemic inflammatory marker associated with aortic remodeling and postoperative outcomes. APTT was adopted to assess coagulation function, which is closely associated with bleeding risk, access site complications and delayed postoperative recovery. These indicators are non-invasive, easily obtainable preoperatively, and widely used in clinical risk stratification.

### Exercise tolerance

6-minute walking distance (6MWD): Conducted in a 30-meter corridor according to American Thoracic Society guidelines (16) within 1–3 days preoperatively; patients walked at maximum tolerable speed for 6 min, and the distance was recorded.

Hand grip strength (HGS): Measured with a digital dynamometer (dominant hand, 3 trials, maximum value recorded) (17).

### Nutritional indicators

Geriatric nutritional risk index (GNRI) (9): Calculated using the formula: GNRI = 14.89  ×   serum albumin (g/dL) + 41.7  ×   (actual body weight/ideal body weight). Ideal body weight was defined as [0.75 ×  height (cm) - 62.5] for males and [0.60 ×  height (cm) - 40] for females.

Prognostic nutritional index (PNI) (18): Calculated using the formula: PNI = 10  ×   serum albumin (g/dL) + 5 ×  lymphocyte count ( ×  10^9^/L).

Serum albumin: Measured within 24 h preoperatively using standard laboratory methods.

### Inflammatory indicators

Neutrophil-to-lymphocyte ratio (NLR): Calculated as neutrophil count divided by lymphocyte count.

Platelet-to-lymphocyte ratio (PLR): Calculated as platelet count divided by lymphocyte count.

High-sensitivity C-reactive protein (hs-CRP): Measured within 24 h preoperatively using immunoassay.

### Coagulation indicators

Prothrombin time-international normalized ratio (PT-INR) and activated partial thromboplastin time (APTT): Measured within 24 h preoperatively using standard coagulation function detection methods. Coagulation indicators were selected because abnormal coagulation increases the risk of perioperative bleeding, access site injury and delayed discharge, which are key prognostic factors for patients undergoing TEVAR (5).

### Other indicators

Demographic characteristics [age, gender, height, weight, body mass index (BMI)], vital signs [systolic blood pressure [SBP], diastolic blood pressure [DBP], heart rate], comorbidities [hypertension, diabetes, smoking, drinking, previous stroke, coronary artery disease (CAD), Tumor], acute complications (uncontrolled hypertension, refractory pain, impending aortic rupture, malperfusion syndrome, pleural effusion), aortic anatomical parameters [distal left common carotid artery [LCCA] diameter, distal left subclavian artery [LSA] diameter, distal descending aorta diameter, LCCA-LSA distance, main trunk proximal/distal diameter], and operative variables [operation duration, contrast agent volume, femoral sheath diameter, type of vascular closure device (ProGlide™/ProStyle™, Perclose)] were extracted from electronic medical records.

### Data collection and follow-up

Clinical data were collected by reviewing electronic medical records, including demographic information, comorbidities, surgical details, laboratory test results, and in-hospital outcomes (access site complication, pneumonia, renal failure). Follow-up was conducted via outpatient visits, telephone interviews, and CTA reexaminations. Information on survival status, discharge disposition, and occurrence of mid-term MAEs was collected during follow-up.

### Statistical analysis

SPSS 26.0 and R 4.5.1 software were used for statistical analysis. Continuous variables were expressed as median [interquartile range (IQR)] due to non-normal distribution (verified by Shapiro–Wilk test) and compared using the Mann–Whitney U test. Categorical variables were expressed as *n* (%) and compared using the *χ*^2^ test or Fisher's exact test. Logistic regression analysis was used to identify factors associated with delayed discharge.

Given the low event counts for delayed discharge and mid-term MAEs, pre-specified core clinical covariates [age, sex, pathology type (TBAD, IMH, PAU), comorbidities, operative variables, and key study indicators] were entered into parsimonious multivariable models to avoid overfitting. Pathology type was adjusted for in all multivariable models to account for heterogeneity across different subtypes of type B aortic pathology. Sensitivity analysis restricted to patients with classic type B aortic dissection (TBAD, *n* = 264) was performed to verify the robustness of the primary results. The proportional-hazards assumption for all Cox regression models was formally tested using Schoenfeld residuals. No violation of the proportional-hazards assumption was detected for any included variable (all *P* > 0.05), confirming that the proportional-hazards assumption was adequately satisfied. Penalized regression approaches were considered for model stability given the limited events. A nomogram for predicting delayed discharge was constructed based on independent predictors identified by multivariate logistic regression. Calibration of the nomogram was evaluated using the Hosmer-Lemeshow test and calibration curve. Discriminative ability was assessed using the receiver operating characteristic (ROC) curve and area under the curve (AUC). Decision curve analysis (DCA) was performed to evaluate the clinical net benefit of the nomogram. Interaction analysis was conducted by including interaction terms (6MWD   ×   GNRI, 6MWD   ×   NLR) in the regression models to explore the combined effects of exercise tolerance with nutritional and inflammatory indicators. The cutoffs for “low” and “high” were defined by the median values of 6MWD, GNRI, and NLR in the study population. Interaction was tested on the multiplicative scale; additive interaction metrics were not calculated in this study. Restricted cubic spline (RCS) analysis with 3 knots was used to evaluate the dose-response relationships between 6MWD/HGS and the two primary outcomes, with *P* for nonlinearity calculated to test the linearity of the associations. A two-sided *P* < 0.05 was considered statistically significant. No missing data were observed for any of the candidate variables in the present study. Therefore, a complete-case analysis was performed for all statistical models. The missing data rate was 0% for all variables included in the univariate and multivariable analyses.

## Results

### Baseline characteristics

**Figure 1:**
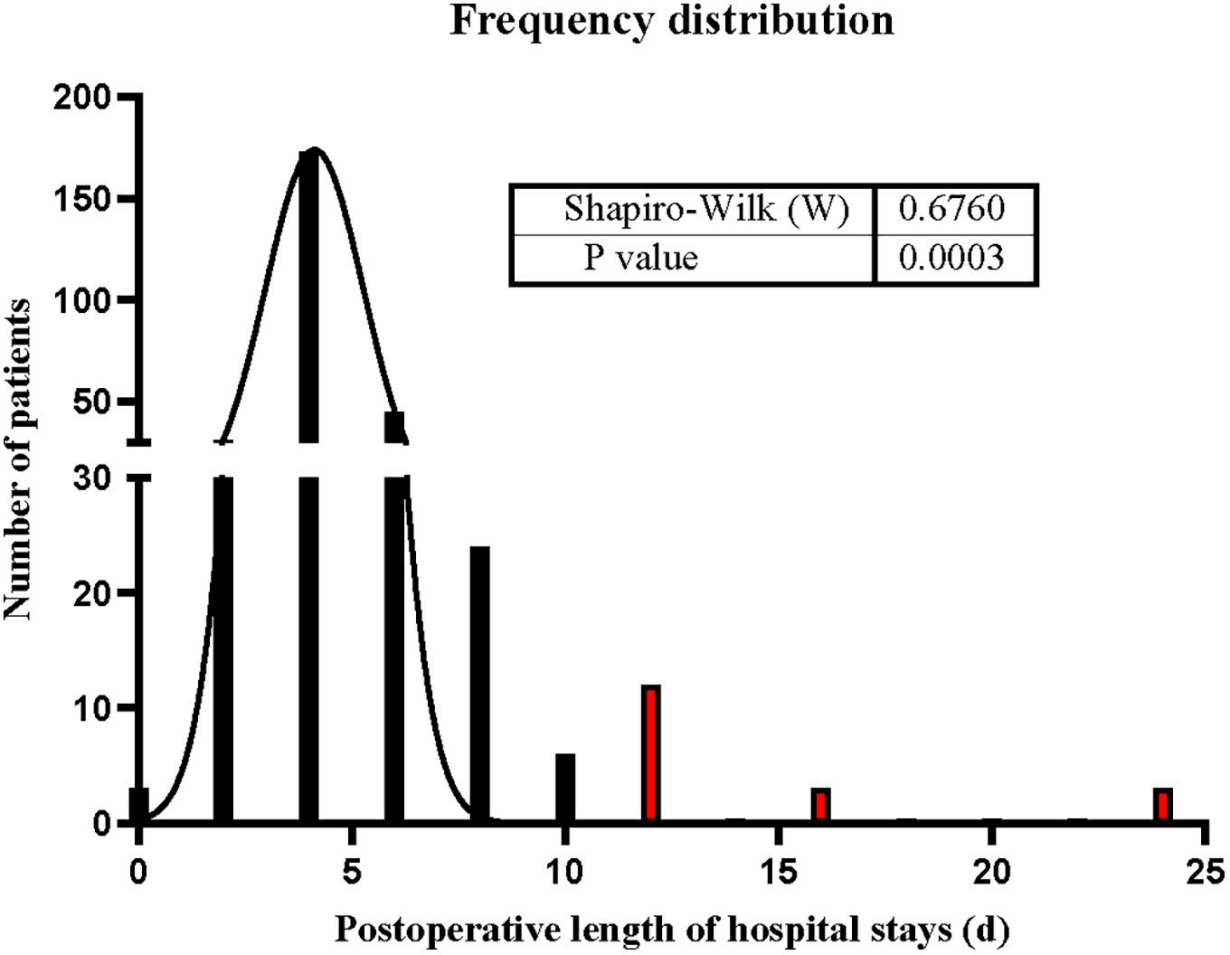
Histogram of postoperative length of hospital stay. Patients with postoperative stay exceeding the 95th percentile (red bars) were defined as having delayed discharge, which was used as one of the primary endpoints in this study.

The histogram of postoperative length of hospital stay (Figure 1) showed a right-skewed distribution, with Shapiro–Wilk test confirming non-normality (W = 0.6760, *P* = 0.0003). 95% of patients were discharged within 10 days after TEVAR. Patients with postoperative stay exceeding the 95th percentile (red bars) were defined as having delayed discharge, which was used as one of the primary endpoints in this study.

A total of 300 patients were enrolled, including 282 in the non-delayed discharge group and 18 in the delayed discharge group. Delayed discharge was defined as postoperative hospital stay >10 days. No significant intergroup differences were observed in demographic indicators (age, sex), vital signs (SBP, DBP, heart rate), type B aortic pathology subtypes (TBAD, IMH, PAU), most comorbidities, clinical symptoms, acute complications, routine laboratory indices (hemoglobin, hematocrit, D-dimer, hs-CRP, WBC), all aortic anatomical parameters, and operative variables (all *P* > 0.05). Among the 300 enrolled patients, 264 (88.0%) were diagnosed with TBAD, 12 (4.0%) with IMH, and 24 (8.0%) with PAU. In total, 208 (69.3%) patients had complicated B-AAS and 92 (30.7%) had uncomplicated B-AAS; 211 (70.3%) received urgent TEVAR and 89 (29.7%) received elective TEVAR. The main drivers for TEVAR were malperfusion syndrome (*n*=48, 16.0%), refractory pain (*n*=77, 25.7%), uncontrolled hypertension (*n*=23, 7.7%), impending rupture (*n*=3, 1.0%), rapid aortic expansion (*n*=57, 19.0%), and high-risk aneurysmal degeneration (*n*=92, 30.7%).

The delayed discharge group had a significantly lower BMI (Z=−3.08, *P*=0.002) and a notably higher incidence of previous stroke (22.22% vs. 3.55%, *P*=0.006) compared with the non-delayed discharge group. For coagulation and nutritional indices, the delayed discharge group presented significantly elevated PT-INR (Z=−3.10, *P*=0.002) and APTT (Z=−2.86, *P*=0.004), along with remarkably decreased albumin (Z=−2.62, *P*=0.009), GNRI (Z=−3.22, *P*=0.001) and PNI (Z=−2.17, *P*=0.030). Inflammatory ratios including NLR (Z=−2.18, *P*=0.029) and PLR (Z=−1.98, *P*=0.047) were significantly higher in the delayed discharge group. Additionally, functional assessment indices including 6MWD (Z=−2.96, *P*=0.003) and HGS (Z=−2.74, *P*=0.006) were significantly lower in the delayed discharge group. Regarding in-hospital outcomes, the delayed discharge group had a significantly higher rate of access site complications (22.22% vs. 1.42%, *P* < 0.001), while the incidence of pneumonia and renal failure did not differ between groups (*P* > 0.05) (Table 1). Further correlation analyses showed that there were no significant associations between other in-hospital complications (pneumonia, renal failure) and preoperative exercise tolerance, nutritional, inflammatory or coagulation indicators (all *P* > 0.05). Further analysis revealed that access site complications were not significantly associated with failure of ProGlide™/ProStyle™ or Perclose devices, nor with femoral sheath dimensions (both *P* > 0.05).

**Table 1:** Baseline characteristics of the study population stratified by delayed discharge status.

Variables	Total (*n* = 300)	Non-delayed discharge (*n* = 282)	Delayed discharge (*n* = 18)	Statistic	*P*
Age, years, M (Q_1_, Q_3_)	52.50 (42.75, 63.25)	52.50 (43.00, 63.00)	54.00 (42.25, 65.25)	Z=−0.37	0.714
BMI, kg/m^2^, M (Q₁, Q₃)	22.43 (20.07, 24.57)	22.56 (20.27, 24.77)	19.49 (18.35, 22.34)	Z=−3.08	0.002
Sex, n(%)				*χ*^2^=1.68	0.195
Male	177 (59.00)	169 (59.93)	8 (44.44)		
Female	123 (41.00)	113 (40.07)	10 (55.56)		
SBP, mmHg, M (Q_1_, Q_3_)	153.00 (140.00, 165.00)	152.50 (140.00, 164.75)	154.00 (138.75, 164.50)	Z=−0.14	0.886
DBP, mmHg, M (Q_1_, Q_3_)	90.00 (80.00, 100.00)	90.00 (80.00, 100.00)	90.50 (80.00, 103.50)	Z=−0.16	0.873
Heart rate, beats/min, M (Q_1_, Q_3_)	80.00 (78.00, 90.00)	80.00 (78.25, 90.00)	86.50 (78.00, 99.00)	Z=−1.10	0.270
Pathology type					
TBAD, n(%)	264 (88.00)	247 (87.59)	17 (94.44)	χ^2^=0.24	0.621
IMH, n(%)	12 (4.00)	12 (4.26)	0 (0.00)	-	1.000
PAU, n(%)	24 (8.00)	23 (8.16)	1 (5.56)	χ^2^=0.00	1.000
Comorbidity					
Hypertension, n(%)	194 (64.67)	182 (64.54)	12 (66.67)	χ^2^=0.03	0.855
CAD, n(%)	30 (10.00)	29 (10.28)	1 (5.56)	χ^2^=0.06	0.808
Previous stroke, n(%)	14 (4.67)	10 (3.55)	4 (22.22)	-	0.006
Diabetes, n(%)	14 (4.67)	14 (4.96)	0 (0.00)	-	1.000
Tumor, n(%)	9 (3.00)	7 (2.48)	2 (11.11)	-	0.095
Smoking, n(%)	138 (46.00)	130 (46.10)	8 (44.44)	χ^2^=0.02	0.891
Drinking, n(%)	27 (9.00)	23 (8.16)	4 (22.22)	χ^2^=2.55	0.110
Sudden chest pain, n(%)	171 (57.00)	164 (58.16)	7 (38.89)	χ^2^=2.56	0.109
Acute complication					
Uncontrolled hypertension, n(%)	23 (7.67)	23 (8.16)	0 (0.00)	χ^2^=0.65	0.421
Refractory pain, n(%)	77 (25.67)	76 (26.95)	1 (5.56)	χ^2^=3.02	0.082
Impending aortic rupture, n(%)	3 (1.00)	3 (1.06)	0 (0.00)	-	1.000
Malperfusion syndrome	16 (5.33)	15 (5.32)	1 (5.56)	-	1.000
Extremity malperfusion, n(%)	9 (3.00)	9 (3.19)	0 (0.00)	-	1.000
Renal malperfusion, n(%)	5 (1.67)	4 (1.42)	1 (5.56)	-	0.268
Cardiac malperfusion, n(%)	2 (0.67)	2 (0.71)	0 (0.00)	-	1.000
Pleural effusion, n(%)	33 (11.00)	33 (11.70)	0 (0.00)	χ^2^=1.32	0.250
Laboratory tests					
Hemoglobin, g/dL, M (Q_1_, Q_3_)	12.70 (11.50, 14.20)	12.70 (11.60, 14.20)	12.10 (10.77, 13.55)	Z=−1.16	0.245
Hematocrit, %, M (Q_1_, Q_3_)	37.70 (34.00, 42.02)	37.70 (34.00, 41.95)	36.40 (34.02, 43.90)	Z=−0.04	0.968
D dimer, μg/mL, M (Q_1_, Q_3_)	26.65 (9.88, 82.68)	26.60 (9.80, 83.40)	30.00 (10.30, 78.10)	Z=−0.15	0.882
PT-INR, M (Q_1_, Q_3_)	1.06 (1.00, 1.14)	1.06 (1.00, 1.13)	1.15 (1.08, 1.56)	Z=−3.10	0.002
APTT, s, M (Q_1_, Q_3_)	34.00 (30.37, 39.62)	34.00 (30.22, 38.50)	43.65 (35.90, 45.55)	Z=−2.86	0.004
Albumin, g/dL, M (Q_1_, Q_3_)	3.90 (3.40, 4.20)	3.90 (3.50, 4.20)	3.35 (2.92, 3.95)	Z=−2.62	0.009
GNRI, M (Q_1_, Q_3_)	99.47 (93.07, 107.95)	99.59 (93.68, 108.42)	86.68 (79.50, 105.85)	Z=−3.22	0.001
PNI, M (Q_1_, Q_3_)	103.00 (81.88, 145.12)	103.50 (83.12, 147.00)	77.00 (65.88, 110.00)	Z=−2.17	0.030
WBC, ×10^9^/L, M (Q_1_, Q_3_)	10.63 (7.79, 13.98)	10.53 (7.80, 13.97)	11.25 (7.28, 15.12)	Z=−0.15	0.883
NLR, M (Q_1_, Q_3_)	6.00 (2.97, 9.78)	5.98 (2.86, 9.56)	8.71 (5.12, 14.52)	Z=−2.18	0.029
PLR, M (Q_1_, Q_3_)	198.22 (119.84, 279.04)	187.95 (118.71, 278.67)	237.02 (167.76, 337.50)	Z=−1.98	0.047
hs-CRP, mg/L, M (Q_1_, Q_3_)	23.29 (5.32, 58.43)	22.84 (4.96, 61.13)	31.59 (15.62, 54.31)	Z=−0.88	0.377
6MWD, m, M (Q_1_, Q_3_)	475.90 (419.50, 539.25)	480.00 (428.60, 540.28)	391.10 (345.97, 490.95)	Z=−2.96	0.003
HGS, kg, M (Q_1_, Q_3_)	29.26 (27.05, 31.22)	29.54 (27.86, 31.22)	25.55 (14.98, 29.26)	Z=−2.74	0.006
Aortic measurements					
Distal LCCA aortic diameter, mm, M (Q_1_, Q_3_)	30.55 (28.30, 32.20)	30.55 (28.30, 32.20)	30.65 (28.72, 31.90)	Z=−0.02	0.983
Distal LSA aortic diameter, mm, M (Q_1_, Q_3_)	29.50 (27.50, 31.40)	29.50 (27.50, 31.48)	29.10 (27.85, 31.28)	Z=−0.14	0.892
Distal descending aorta diameter, mm, M (Q_1_, Q_3_)	23.20 (21.10, 25.60)	23.10 (21.10, 25.60)	23.70 (21.55, 26.15)	Z=−0.85	0.396
Distal LSA diameter, mm, M (Q_1_, Q_3_)	9.60 (9.00, 10.15)	9.60 (9.00, 10.00)	9.65 (8.85, 11.20)	Z=−0.89	0.375
LCCA-LSA distance, mm, M (Q_1_, Q_3_)	10.00 (7.00, 14.00)	10.00 (7.00, 14.00)	9.50 (8.00, 16.25)	Z=−0.39	0.697
Main trunk proximal diameter, mm, M (Q_1_, Q_3_)	32.00 (30.00, 34.00)	32.00 (30.00, 34.00)	32.00 (30.00, 34.00)	Z=−0.80	0.422
Main trunk distal diameter, mm, M (Q_1_, Q_3_)	26.00 (24.00, 28.00)	26.00 (24.00, 28.00)	26.00 (24.00, 28.00)	Z=−0.83	0.405
Operative procedure					
Operation duration, min, M (Q_1_, Q_3_)	45.00 (34.00, 60.00)	46.00 (32.50, 60.00)	43.00 (36.00, 47.00)	Z=−0.60	0.549
Contrast agent volume, mL, M (Q_1_, Q_3_)	190.00 (170.00, 200.00)	190.00 (170.00, 200.00)	180.00 (170.00, 190.00)	Z=−1.11	0.269
In-hospital outcomes					
Access site complication, n(%)	8 (2.67)	4 (1.42)	4 (22.22)	-	<0.001
Pneumonia, n(%)	3 (1.00)	3 (1.06)	0 (0.00)	-	1.000
Renal failure, n(%)	2 (0.67)	1 (0.35)	1 (5.56)	-	0.117

BMI, body mass index; 6MWD, 6-minute walking distance; HGS, hand grip strength; GNRI, geriatric nutritional risk index; PNI, prognostic nutritional index; NLR, neutrophil-to-lymphocyte ratio; PLR, platelet-to-lymphocyte ratio; hs-CRP, high-sensitivity C-reactive protein.

### Predictors of delayed discharge

Univariate and multivariate logistic regression analyses identified preoperative predictors of delayed discharge post-TEVAR (Table 2). Univariate analysis showed that elevated APTT, elevated NLR, decreased GNRI, decreased 6MWD, and decreased HGS were significantly associated with delayed discharge (all *P* < 0.05). Multivariate analysis confirmed these five variables as independent preoperative predictors of delayed discharge: elevated APTT (OR=1.01, *P*=0.043), decreased GNRI (OR=0.93, *P*=0.002), elevated NLR (OR=1.07, *P*=0.013), decreased 6MWD (OR=0.99, *P*=0.026), and decreased HGS (OR=0.92, *P*=0.025).

**Table 2:** Univariate and multivariate logistic regression analysis of factors associated with delayed discharge.

Variables	Univariate analysis					Multivariate analysis				
	*β*	S.E	Z	*P*	OR (95%CI)	β	S.E	Z	*P*	OR (95%CI)
Pathology Type										
TBAD					1.00 (Reference)					
IMH	−14.89	1142.05	−0.01	0.990	0.00 (0.00∼Inf)					
PAU	−0.46	1.05	−0.44	0.662	0.63 (0.08∼4.96)					
Hypertension	0.09	0.52	0.18	0.855	1.10 (0.40∼3.02)					
CAD	−0.67	1.05	−0.64	0.524	0.51 (0.07∼4.00)					
Previous stroke	2.05	0.65	3.14	0.002	7.77 (2.17∼27.89)					
Tumor	1.59	0.84	1.89	0.059	4.91 (0.94∼25.58)					
Smoking	−0.07	0.49	−0.14	0.891	0.94 (0.36∼2.44)					
Drinking	1.17	0.61	1.92	0.054	3.22 (0.98∼10.58)					
Sudden chest pain	−0.78	0.50	−1.57	0.117	0.46 (0.17∼1.22)					
Refractory pain	−1.84	1.04	−1.77	0.077	0.16 (0.02∼1.22)					
Malperfusion syndrome	0.05	1.06	0.04	0.965	1.05 (0.13∼8.40)					
Sex										
Female					1.00 (Reference)					
Male	0.63	0.49	1.28	0.201	1.87 (0.72∼4.88)					
Age	0.01	0.02	0.35	0.730	1.01 (0.97∼1.04)					
SBP	−0.01	0.01	−0.48	0.634	0.99 (0.97∼1.02)					
DBP	−0.01	0.02	−0.40	0.689	0.99 (0.96∼1.03)					
Heart rate	0.04	0.02	1.73	0.083	1.04 (1.00∼1.08)					
Distal LCCA aortic diameter	0.02	0.09	0.21	0.832	1.02 (0.86∼1.21)					
Distal LSA aortic diameter	−0.02	0.09	−0.20	0.842	0.98 (0.82∼1.18)					
Distal descending aorta diameter	0.10	0.07	1.46	0.145	1.11 (0.97∼1.27)					
Distal LSA diameter	0.17	0.11	1.52	0.128	1.18 (0.95∼1.46)					
LCCA LSA distance	0.02	0.04	0.39	0.696	1.02 (0.94∼1.10)					
Main trunk proximal diameter	0.10	0.09	1.02	0.308	1.10 (0.92∼1.32)					
Main trunk distal diameter	0.10	0.09	1.05	0.294	1.10 (0.92∼1.33)					
Operation duration	−0.01	0.01	−0.88	0.378	0.99 (0.97∼1.01)					
Contrast agent volume	−0.02	0.01	−1.49	0.136	0.98 (0.96∼1.01)					
Hemoglobin	−0.14	0.12	−1.18	0.240	0.87 (0.70∼1.10)					
Hematocrit	0.04	0.03	1.57	0.116	1.04 (0.99∼1.10)					
D dimer	−0.00	0.00	−0.25	0.799	1.00 (0.99∼1.00)					
PT INR	0.00	0.07	0.03	0.976	1.00 (0.88∼1.14)					
APTT	0.01	0.00	2.14	0.032	1.01 (1.01∼1.02)	0.01	0.01	2.03	0.043	1.01 (1.01∼1.02)
GNRI	−0.09	0.02	−3.82	<0.001	0.91 (0.87∼0.96)	−0.08	0.02	−3.08	0.002	0.93 (0.88∼0.97)
WBC	0.05	0.05	1.13	0.260	1.05 (0.96∼1.15)					
NLR	0.05	0.02	2.46	0.014	1.06 (1.01∼1.10)	0.06	0.03	2.48	0.013	1.07 (1.01∼1.12)
PLR	0.00	0.00	1.90	0.058	1.00 (1.00∼1.00)					
CRP	−0.00	0.00	−0.06	0.952	1.00 (0.99∼1.01)					
6MWD	−0.01	0.00	−3.22	0.001	0.99 (0.99∼0.99)	−0.01	0.00	−2.23	0.026	0.99 (0.99∼0.99)
HGS	−0.10	0.03	−3.18	0.001	0.90 (0.85∼0.96)	−0.08	0.04	−2.25	0.025	0.92 (0.86∼0.99)
PNI	−0.02	0.01	−2.72	0.007	0.98 (0.96∼0.99)					

### Nomogram for predicting delayed discharge

**Figure 2:**
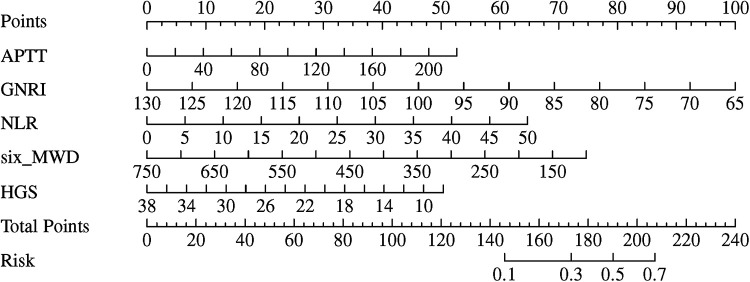
Nomogram for predicting delayed discharge after TEVAR in patients with type B aortic dissection. This nomogram was constructed using five independent predictors from the multivariate model: APTT, GNRI, NLR, 6MWD, and HGS. To use it, locate each predictor’s value on its axis, draw a vertical line to the top “Points” scale to get assigned points, sum these to get total points, then project the total to the bottom “Risk” scale to determine the predicted probability of delayed discharge.

A preoperative nomogram was developed to predict the probability of delayed discharge after TEVAR, incorporating five independent preoperative predictors identified in the multivariate logistic regression analysis: APTT, GNRI, NLR, 6MWD, and HGS (Figure 2). To use the nomogram, the value of each predictor is located on its corresponding axis, and a vertical line is drawn to the top “Points” scale to obtain the assigned points. The total points are calculated by summing the points from all predictors, and a vertical line is drawn from the total points to the bottom “Risk” scale to determine the predicted probability of delayed discharge.

**Figure 3:**
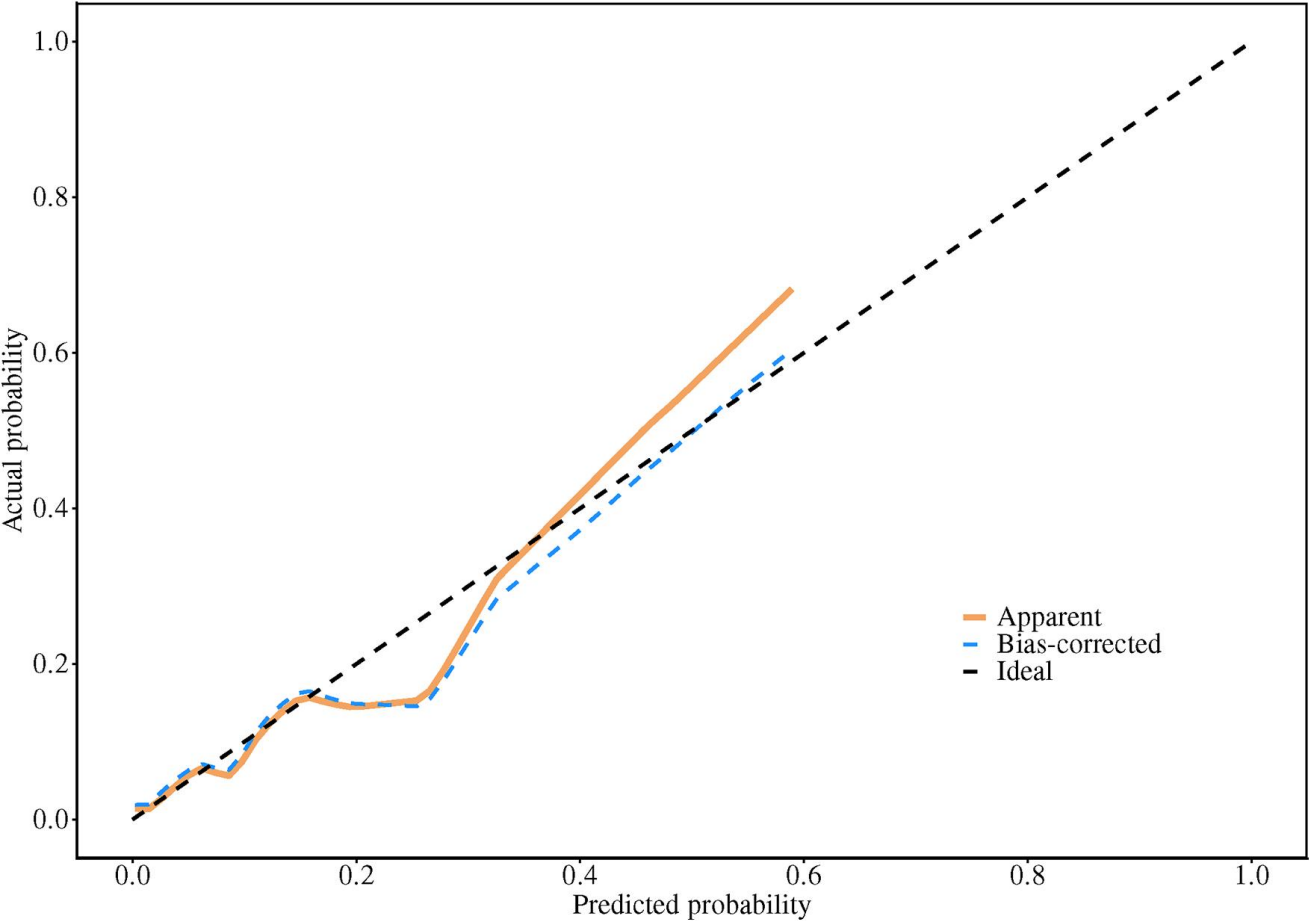
Calibration curve of the nomogram for predicting delayed discharge after TEVAR.

The calibration curve of the nomogram was plotted to assess the agreement between the predicted and observed probabilities of delayed discharge (Figure 3). The model demonstrated good calibration, with the predicted probabilities closely aligning with the actual probabilities across the entire risk spectrum, and the Hosmer-Lemeshow test indicated no significant departure from perfect calibration (*χ*^2^ = 8.32, *P* = 0.403).

**Figure 4:**
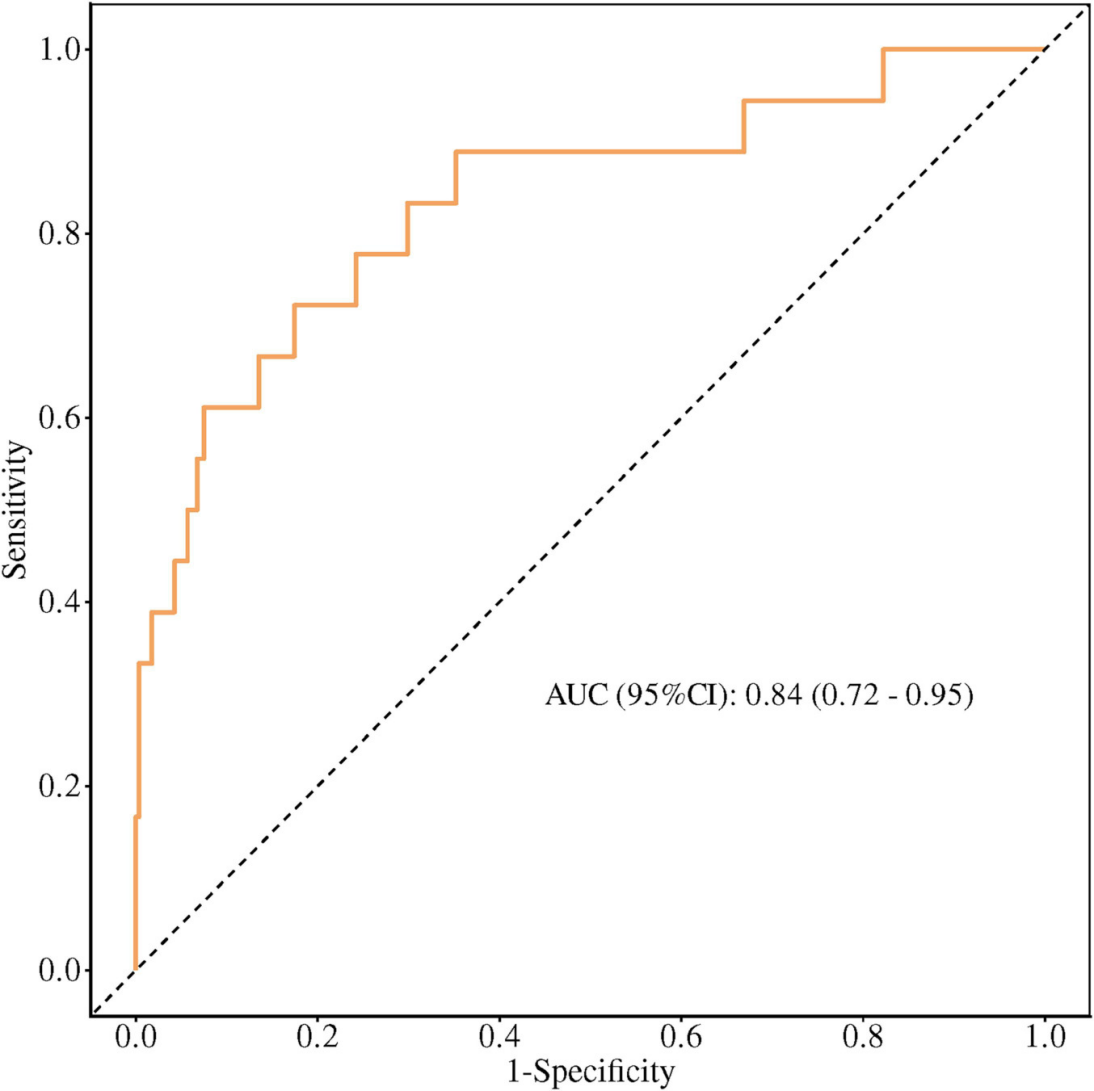
Receiver operating characteristic (ROC) curve of the nomogram for predicting delayed discharge after TEVAR.

The ROC curve was generated to evaluate the discriminative ability of the nomogram (Figure 4). The area under the ROC curve (AUC) was 0.84 (95% confidence interval: 0.72−0.95), indicating excellent discriminatory performance in identifying patients at high risk of delayed discharge. The optimal cutoff value of the predicted probability was 0.144, with a sensitivity of 83.3% and a specificity of 82.6% for predicting delayed discharge.

**Figure 5:**
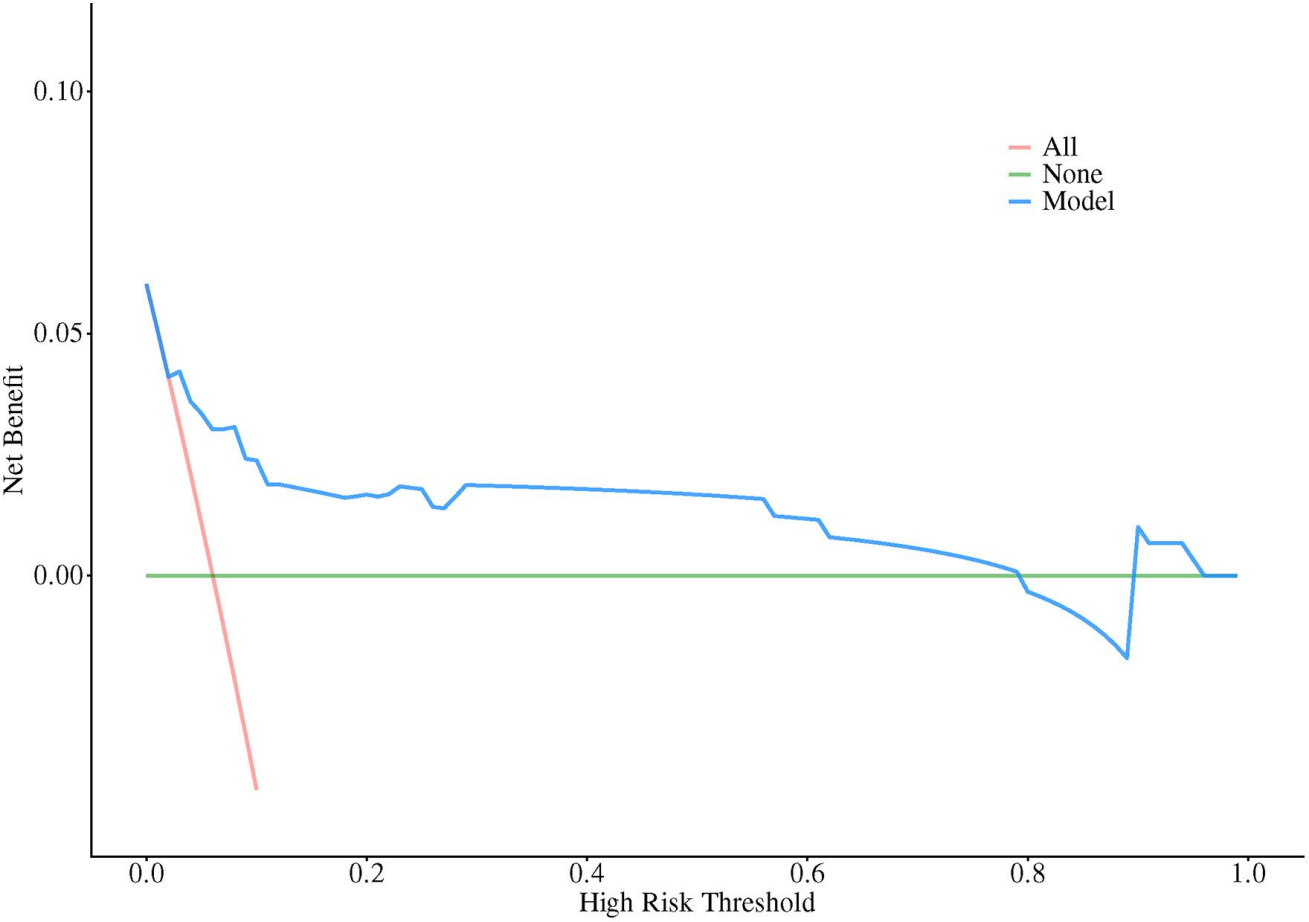
DCA of the nomogram for predicting delayed discharge after TEVAR.

Decision curve analysis (DCA) was performed to assess the clinical net benefit of the nomogram across a range of high-risk thresholds (Figure 5). The model curve (blue) lay above both the “None” (green) and “All” (red) reference lines across most clinically relevant threshold probabilities, indicating that the nomogram provides a favorable net benefit and can be used to support personalized clinical decision-making for patients at high risk of delayed discharge.

This visual and quantitative tool enables clinicians to stratify the risk of delayed discharge before TEVAR and implement targeted perioperative interventions. Model coefficients, intercept, and predicted-risk equation for the nomogram: Logit(P) = 0.01  ×  APTT−0.06  ×  GNRI + 0.07  ×  NLR−0.01 × 6MWD−0.07 × HGS−6.82. Predicted risk of delayed discharge = 1/{1 + exp[−Logit(P)]}, where *P* = probability of delayed discharge and 6.82 is the model intercept.

### Predictors of mid-term adverse events

During a median follow-up of 485 days (IQR: 405–894 days), mid-term adverse events occurred in 38 patients (12.7%). These events were classified as components of mid-term MAEs. Mid-term MAEs included spinal cord ischemia (SCI) in 4 patients (1.3%), acute myocardial infarction (AMI) in 2 patients (0.7%), endoleak in 20 patients (6.7%), stenosis of the branched section in 9 patients (3.0%), and progressive aortic dilatation in 3 patients (1.0%). Re-intervention was mainly performed for the management of endoleak and progressive aortic dilatation. No mid-term mortality was observed.

Univariate and multivariate Cox regression analyses identified predictors of mid-term MAEs post-TEVAR (Table 3). Univariate analysis showed that drinking, older age, elevated heart rate, larger distal LSA diameter, longer LCCA-LSA distance, shorter operation duration, decreased GNRI and HGS, as well as elevated NLR and reduced 6MWD were significantly associated with mid-term MAEs (all *P* < 0.05). Multivariate analysis confirmed nine independent predictors of mid-term MAEs: drinking (HR=9.73, *P* < 0.001), increasing age (HR=0.95, *P*=0.015), elevated heart rate (HR=1.05, *P*=0.022), longer LCCA-LSA distance (HR=1.19, *P* < 0.001), shorter operation duration (HR=0.95, *P*=0.003), decreased GNRI (HR=0.84, *P* < 0.001), elevated NLR (HR=1.03, *P*=0.037), increased 6MWD (HR=1.01, *P*=0.022), and decreased HGS (HR=0.87, *P* < 0.001). Of note, distal LSA diameter failed to remain an independent predictor in the multivariate model (*P*=0.095).

**Table 3:** Univariate and multivariate Cox regression analysis of factors associated with mid-term MAEs.

Variables	Univariate Cox analysis					Multivariate Cox analysis				
	β	S.E	Z	*P*	HR (95%CI)	β	S.E	Z	*P*	HR (95%CI)
Drinking	2.02	0.34	5.89	<0.001	7.53 (3.84∼14.74)	2.28	0.47	4.81	<0.001	9.73 (3.85∼24.56)
Age	−0.03	0.01	−2.60	0.009	0.97 (0.94∼0.99)	−0.05	0.02	−2.44	0.015	0.95 (0.92∼0.99)
Heart rate	0.03	0.01	2.37	0.018	1.03 (1.01∼1.06)	0.05	0.02	2.29	0.022	1.05 (1.01∼1.09)
Distal LSA diameter	0.14	0.06	2.25	0.024	1.15 (1.02∼1.31)	0.26	0.16	1.67	0.095	1.30 (0.96∼1.76)
LCCA LSA distance	0.06	0.02	2.91	0.004	1.06 (1.02∼1.10)	0.18	0.04	4.30	<0.001	1.19 (1.10∼1.29)
Operation duration	−0.02	0.01	−2.16	0.031	0.98 (0.96∼0.99)	−0.05	0.02	−2.95	0.003	0.95 (0.92∼0.98)
GNRI	−0.16	0.02	−9.77	<0.001	0.85 (0.83∼0.88)	−0.17	0.03	−6.39	<0.001	0.84 (0.80∼0.89)
NLR	0.03	0.02	2.06	0.039	1.03 (1.01∼1.07)	0.01	0.01	2.15	0.037	1.03 (1.01∼1.06)
6MWD	−0.01	0.00	−2.02	0.043	0.99 (0.99∼0.99)	0.01	0.00	2.29	0.022	1.01 (1.01∼1.01)
HGS	−0.11	0.02	−5.34	<0.001	0.90 (0.86∼0.93)	−0.14	0.03	−4.28	<0.001	0.87 (0.81∼0.93)
PNI	−0.01	0.00	−2.83	0.005	0.99 (0.98∼0.99)	−0.01	0.01	−1.75	0.080	0.99 (0.98∼1.00)

Multivariable Cox regression models were adjusted for sex and pathology type (TBAD, IMH, PAU). All variables listed in the table were entered into the univariate analysis first; statistically significant variables were further included in the multivariate analysis. HGS was adjusted for sex, age, and body size.

### Interaction analysis

**Figure 6:**
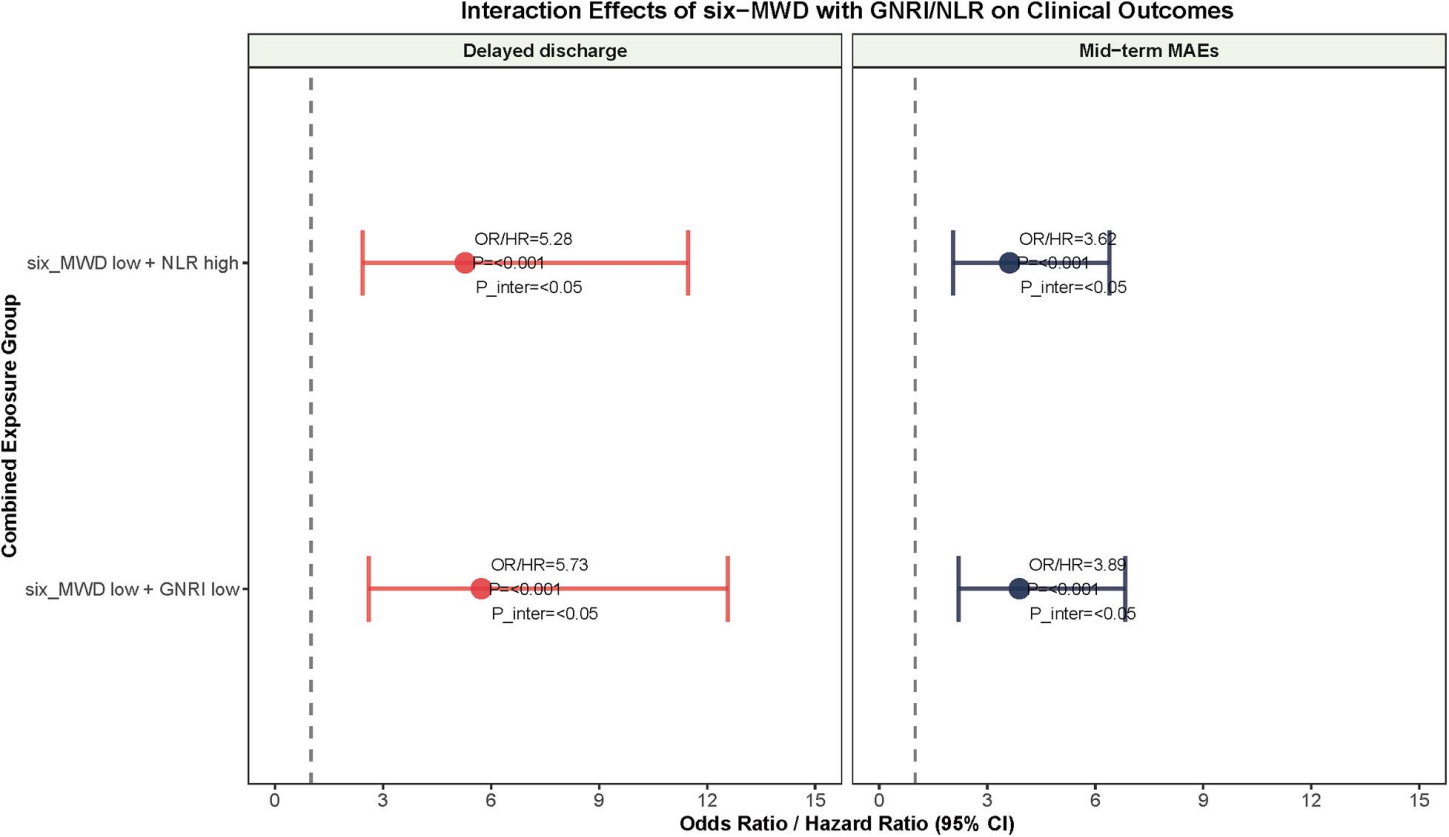
Interaction analysis of six_MWD with GNRI/NLR on clinical outcomes. All subgroups were dichotomized by median value: low 6MWD, low GNRI, and high NLR. Reference categories were defined as high 6MWD + high GNRI for GNRI stratification, and high 6MWD + low NLR for NLR stratification. Odds ratios are presented for delayed discharge, and hazard ratios are presented for mid-term major adverse events.

Using median-based cutoffs, low 6MWD combined with low GNRI significantly increased the risk of delayed discharge (OR=5.73, 95%CI:2.61–12.58, *P* < 0.001) and mid-term MAEs (HR=3.89, 95%CI:2.21–6.83, *P* < 0.001) (Figure 6). Low 6MWD combined with high NLR also significantly elevated the risk of delayed discharge (OR=5.28, 95%CI:2.43–11.47, *P* < 0.001) and mid-term MAEs (HR=3.62, 95%CI:2.05–6.39, *P* < 0.001) (*P* for interaction <0.05 for all).

### Dose-response relationship

**Figure 7:**
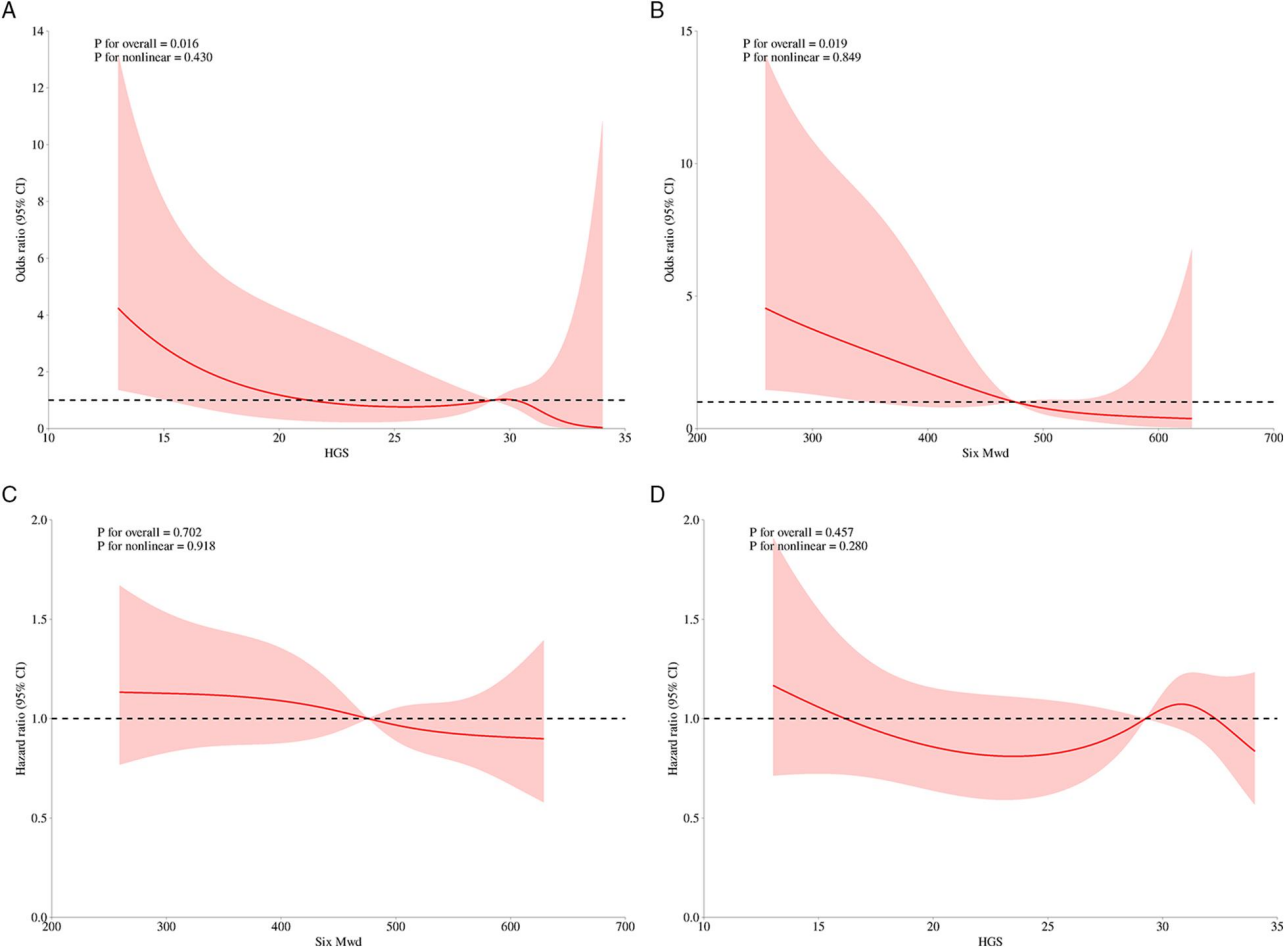
Dose-response relationships between HGS/6MWD and risks of delayed discharge and mid-term MAEs based on restricted cubic spline analysis. **(A)** Adjusted odds ratios (ORs) for delayed discharge according to HGS; **(B)** adjusted ORs for delayed discharge according to 6MWD; **(C)** adjusted hazard ratios (HRs) for mid-term MAEs according to 6MWD; **(D)** adjusted HRs for mid-term MAEs according to HGS. The solid red lines represent the adjusted effect estimates, with the pink shaded areas indicating the corresponding 95% confidence intervals. The horizontal dashed line denotes the null effect (OR/HR = 1). *P* values for the overall effect and for nonlinearity are presented for each association. All models were adjusted for age and hypertension.

To further quantify the dose-response associations, we plotted the odds ratios (ORs) for delayed discharge and hazard ratios (HRs) for mid-term MAEs across the distributions of HGS and 6MWD using RCS analysis (Figure 7). The overall effects of HGS and 6MWD on delayed discharge were statistically significant (*P* for overall = 0.016 and 0.019, respectively), whereas no significant overall associations were observed for mid-term MAEs (*P* for overall = 0.702 and 0.457, respectively). Importantly, the test for nonlinearity confirmed that all associations followed a linear pattern (*P* for nonlinearity = 0.430, 0.549, 0.918, and 0.280 for HGS-delayed discharge, 6MWD-delayed discharge, 6MWD-MAEs, and HGS-MAEs, respectively). Collectively, these results corroborated that higher levels of 6MWD and HGS were linearly associated with reduced risks of the primary outcomes.

### Sensitivity analysis

Two sensitivity analyses were conducted to confirm the robustness of the primary findings. First, analysis restricted to the 264 patients with classic TBAD yielded results highly consistent with the main analysis: elevated APTT and NLR, as well as decreased GNRI, 6MWD, and HGS remained independent predictors of delayed discharge, and the predictors of mid-term MAEs were also unchanged (all *P* < 0.05). Second, sensitivity analysis using sex-specific HGS categories showed that low sex-stratified HGS remained an independent predictor of both delayed discharge and mid-term MAEs (all *P* < 0.05). These findings confirm that the primary results were robust after accounting for pathological heterogeneity and the sex dependence of grip strength.

## Discussion

This study explored the predictive value of preoperative exercise tolerance (assessed by 6MWD and HGS) and its synergistic effects with nutritional/inflammatory indicators for delayed discharge and mid-term MAEs in patients with type B aortic pathology undergoing TEVAR. Our study focused on patients with TBAD undergoing TEVAR rather than those with aortic aneurysm. TBAD is characterized by acute systemic inflammation and nutritional disturbances, which can significantly reduce exercise tolerance even in young patients, thus making exercise tolerance a valuable prognostic indicator for this population (7, 13). Results confirmed preoperative low exercise tolerance as an independent predictor of both outcomes, with significant synergistic risk when combined with low GNRI or high NLR. RCS analysis verified a linear dose-response relationship (no threshold effect). A well-performing nomogram for delayed discharge was constructed, and multiple independent predictors for mid-term MAEs (including drinking, anatomical and laboratory indicators) were identified. These findings enrich the post-TEVAR risk stratification system and provide a clear basis for individualized perioperative management and prognostic intervention.

### Exercise tolerance and delayed discharge

Delayed discharge is a direct reflection of the body's ability to tolerate surgical stress and recover perioperative function, and is also an important indicator of short-term surgical prognosis and medical resource consumption (3, 15). In this study, the 95th percentile of postoperative stay was 10 days, with a delayed discharge rate of 6.0% (18/300), consistent with previous reports (4, 19). This definition is consistent with the clinical practice of using the 95th percentile to define delayed discharge in vascular surgery (15), and the incidence is in line with the short-term recovery characteristics of TEVAR, a minimally invasive procedure.

Baseline characteristic analysis found no significant differences between the delayed discharge and non-delayed discharge groups in demographic indicators, vital signs, pathology type, most comorbidities, acute complications, aortic anatomical parameters and operative variables, indicating that the two groups had good baseline balance and the influencing factors of delayed discharge were more concentrated in nutritional, inflammatory and functional assessment indicators. Specifically, the delayed discharge group had significantly lower BMI, 6MWD and HGS, and a notably higher incidence of previous stroke; coagulation indices (PT-INR, APTT) were significantly elevated, nutritional indices (albumin, GNRI, PNI) were significantly decreased, and inflammatory ratios (NLR, PLR) were significantly increased.

Univariate and multivariate logistic regression analyses further clarified the independent preoperative predictors of delayed discharge: elevated APTT (OR=1.01), elevated NLR (OR=1.07), decreased GNRI (OR=0.93), decreased 6MWD (OR=0.99) and decreased HGS (OR=0.92). Among them, low 6MWD and low HGS, as core indicators of exercise tolerance, were both confirmed as independent risk factors, which fully indicated that impaired preoperative cardiopulmonary reserve and skeletal muscle strength are key factors leading to delayed postoperative recovery. Exercise tolerance is a comprehensive indicator integrating cardiopulmonary function, skeletal muscle metabolism and systemic physiological reserve (13); TBAD patients with low 6MWD and HGS often have insufficient compensatory capacity, which not only slows down the recovery of basic bodily functions after surgery, but also increases the risk of perioperative complications such as access site abnormalities, and ultimately leads to prolonged hospital stay (19, 20). Consistent with this, our study found that low GNRI is another independent predictor of delayed discharge, and the combination of low 6MWD and low GNRI further increases the risk, highlighting the synergistic effect of nutrition and exercise tolerance on postoperative recovery.

From a mechanistic perspective, the interaction between exercise tolerance and nutrition in affecting delayed discharge is hypothetically mediated by skeletal muscle function and physiological reserve. Poor nutritional status (low GNRI) reduces muscle protein synthesis and induces sarcopenia, resulting in decreased hand grip strength (low HGS) (21). Skeletal muscle mass and function are well-established prognostic markers in aortic surgery: reduced psoas muscle area and lean muscle mass are independently associated with higher complication rates and prolonged hospital stay after aortic endovascular and open surgery (22, 23). Low exercise tolerance (low 6MWD) and poor skeletal muscle function reflect insufficient physiological reserve, which impairs postoperative recovery and increases the risk of in-hospital complications, ultimately leading to delayed discharge. These findings are consistent with our observed data and clinical observations, rather than being supported by direct molecular measurements.

In addition, elevated APTT in the delayed discharge group indicates abnormal coagulation function, which not only increases the risk of bleeding-related access site complications, but also delays wound healing, forming a vicious circle with poor nutrition and low exercise tolerance, and jointly leading to delayed discharge.

To improve the clinical applicability of the research results, this study constructed a preoperative nomogram for predicting delayed discharge incorporating the five aforementioned independent preoperative predictors. The model showed excellent discriminatory ability (AUC=0.84, 95%CI:0.72–0.95) and good calibration (Hosmer-Lemeshow test, *χ*2=8.32, *P*=0.403), and DCA confirmed its favorable clinical net benefit. This visual quantitative tool can help clinicians quickly stratify the risk of delayed discharge before TEVAR, and implement targeted perioperative intervention measures for high-risk patients. Given the narrow preoperative window in acute TEVAR, routine preoperative prehabilitation is not clinically feasible; interventions should focus on perioperative optimization and postoperative rehabilitation.

### Exercise tolerance and mid-term MAEs

Mid-term MAEs (occurring at 6 months to 2 years after TEVAR) are key factors affecting long-term survival of TBAD patients after TEVAR (5, 6). This study showed that low 6MWD and low HGS are independent predictors of mid-term MAEs, which is consistent with findings in other cardiovascular diseases (14, 21). The mechanism may be that low exercise tolerance indicates compromised physiological reserve, making patients less able to cope with postoperative hemodynamic changes, inflammatory responses, and aortic wall remodeling (22). Additionally, TBAD patients with low exercise tolerance often have chronic inflammation and poor nutritional status, which can accelerate aortic wall degradation, increase the risk of endoleak and aortic rupture, and promote the development of cardiovascular events (7, 23). Our study confirmed that high NLR and low GNRI are also independent predictors of mid-term MAEs, and the combination of low exercise tolerance with these factors significantly increases the risk, suggesting that exercise tolerance, nutrition, and inflammation jointly affect mid-term prognosis.

It is worth noting that multivariate analysis showed that increased 6MWD was an independent protective factor for mid-term MAEs (HR=1.01, *P*=0.029), which was consistent with the conclusion of RCS analysis that higher 6MWD is linearly associated with reduced risk of mid-term MAEs. Although the overall effect of 6MWD and HGS on mid-term MAEs was not statistically significant in RCS analysis (*P* for overall=0.702 and 0.457, respectively), the linear dose-response relationship was clear (all *P* for nonlinearity > 0.05), indicating that there is no threshold for the protective effect of exercise tolerance on mid-term prognosis—any improvement in preoperative 6MWD and HGS may help reduce the risk of mid-term MAEs. This finding is of great guiding significance for clinical prehabilitation, suggesting that even moderate exercise intervention for TBAD patients before surgery can bring long-term prognostic benefits.

The interaction analysis further found that low 6MWD combined with high NLR significantly increased the risk of mid-term MAEs (HR=3.62), which reveals the synergistic mechanism of exercise tolerance and inflammatory load on long-term prognosis. The synergistic effect of low exercise tolerance, high inflammatory load (high NLR), and poor nutrition (low GNRI) on mid-term MAEs is biologically plausible but remains a mechanistic hypothesis (these molecular and cellular pathways were not directly measured in this study). Clinically, low 6MWD and HGS indicate impaired physiological reserve, which reduces the ability to adapt to postoperative hemodynamic changes and persistent inflammation (24–26). Our data confirm that low exercise tolerance combined with high NLR or low GNRI independently increases the risk of mid-term MAEs, which is consistent with the prognostic value of skeletal muscle dysfunction in aortic surgery (20, 27–29). These findings are based on our actual observational data and provide a practical basis for clinical risk stratification, rather than on mechanistic measurements.

In addition, the study found that shorter operation duration was an independent predictor of mid-term MAEs (HR=0.94), which may be related to the clinical operation characteristics: overly short operation duration may mean insufficient intraoperative evaluation and stent graft placement, which increases the risk of postoperative complications such as endoleak and stenosis of the branched section. This finding reminds clinicians that TEVAR should pursue the balance between surgical efficiency and operation quality, and avoid the increase of mid-term adverse events caused by hasty operation. Notably, increasing age was associated with a lower risk of mid-term MAEs in the present study. This seemingly paradoxical result is frequently observed in real-world acute aortic syndrome cohorts. Younger patients in our study were more likely to present with uncontrolled hypertension, heavy alcohol use, heavy smoking, and more aggressive aortic wall degeneration, all of which contribute to higher mid-term risks of endoleak, aortic remodeling failure, and reintervention. By contrast, elderly patients were selected for TEVAR under more stringent risk criteria and showed better compliance with postoperative antihypertensive therapy and imaging surveillance. Therefore, the inverse association between age and mid-term MAEs reflects the different risk profiles between younger and older patients in this cohort, rather than a genuine protective effect of advanced age.

### Clinical implications

The findings of this study have important clinical implications. First, preoperative assessment of exercise tolerance (6MWD and HGS), combined with nutritional (GNRI), inflammatory (NLR), and coagulation (APTT) indicators, enables comprehensive risk stratification for TBAD patients undergoing TEVAR, facilitating early identification of high-risk individuals (low 6MWD/HGS with low GNRI or high NLR) who require intensive perioperative monitoring. The constructed nomogram further supports personalized prediction of delayed discharge risk, while targeted preoperative interventions—including nutritional support (e.g., protein/calorie supplementation), anti-inflammatory therapy, lifestyle modifications (smoking cessation, strict alcohol abstinence), coagulation correction, and individualized stent selection/surgical planning for adverse anatomical parameters—may improve physiological reserve (24). Second, postoperative rehabilitation programs tailored to preoperative exercise tolerance (e.g., progressive walking and resistance training) promote functional recovery, reduce delayed discharge, and enhance long-term outcomes (25), with persistent training recommended given the incremental prognostic benefits. Third, the linear dose-response relationship between exercise tolerance and adverse outcomes highlights the prognostic value of functional assessment. Preoperative prehabilitation is not broadly feasible for urgent/acute TEVAR due to the extremely short preoperative window and clinical instability in acute type B aortic pathology. Thus, we only recommend postoperative rehabilitation tailored to exercise tolerance to improve functional recovery and mid-term outcomes. Prehabilitation may be considered solely for elective TEVAR cases with a sufficient preoperative window. Additionally, long-term follow-up should emphasize modifiable risk factors (especially alcohol abstinence), regular imaging reexaminations for aortic diameter and stent status (with early intervention for endoleak or progressive dilatation), and hemodynamic management for patients with elevated heart rate. These simple, non-invasive indicators (6MWD, HGS, GNRI, NLR) can be easily integrated into routine preoperative risk assessment for vascular surgeons, enabling early identification of high-risk patients and targeted interventions to improve postoperative recovery and mid-term outcomes.

### Limitations

This study has several limitations that need to be acknowledged, and these limitations also point out the direction for future research. First, this is a single-center retrospective cohort study, and there may be potential selection bias and residual confounding factors despite strict inclusion and exclusion criteria and multivariate regression adjustment; the sample size is 300 cases, and multicenter studies with larger sample sizes are needed to verify the research results and improve the external validity of the conclusion. Second, exercise tolerance was only assessed preoperatively, and the dynamic changes of 6MWD and HGS during postoperative follow-up were not monitored, so the impact of postoperative exercise tolerance improvement on short-term and long-term prognosis could not be evaluated; future prospective studies can track the dynamic changes of exercise tolerance and explore the causal relationship between exercise intervention and prognosis. Third, this study did not explore the specific molecular mechanisms underlying the association between exercise tolerance and outcomes, and the synergistic mechanism of exercise tolerance, nutrition and inflammation on the aortic wall and vascular endothelial function needs to be further clarified through basic experimental research and clinical translational research. Fourth, the follow-up time of this study is a median of 485 days (about 16 months), which is a mid-term follow-up. We only focused on mid-term MAEs without further time stratification (periprocedural, 30-day, 6-month, etc.) to ensure sufficient statistical power, as short-term adverse events were adequately reflected by the endpoint of delayed discharge. A longer-term follow-up and more detailed time-stratified analysis may be conducted in future studies. Fifth, the study did not consider the influence of postoperative rehabilitation measures and compliance on outcomes, and these factors may have an important impact on the recovery of exercise tolerance and prognosis, which need to be included in the research variables in future studies. Sixth, detailed intraoperative data including intravascular ultrasound (IVUS) application, intraoperative adjunctive procedures, vascular closure device usage and sheath dimensions were not systematically collected and analyzed. As reported by Di Girolamo et al. (30), IVUS guidance can significantly reduce the necessity of intraoperative adjunctive procedures and optimize the intraoperative course in TEVAR for TBAD, which may exert a critical impact on postoperative recovery and prognosis. The lack of these intraoperative strategy-related data may lead to residual confounding, and future studies should incorporate detailed intraoperative parameters to further refine the risk stratification system.

## Conclusions

This study identifies and validates key preoperative predictors of delayed discharge and mid-term MAEs in TBAD patients after TEVAR, highlighting preoperative low exercise tolerance (assessed by 6MWD and HGS) as an independent predictor with a linear dose-response relationship (no threshold effect) for both outcomes. Low exercise tolerance combined with poor nutritional status (low GNRI) or high inflammatory load (high NLR) exerts a significant synergistic risk-increasing effect, while a preoperative nomogram constructed from independent preoperative predictors (elevated APTT/NLR, decreased GNRI/6MWD/HGS) shows good predictive value for delayed discharge. The findings provide a reliable reference for clinicians to stratify perioperative and mid-term risks, enabling early identification of high-risk patients. Individualized preoperative optimization, perioperative monitoring, and postoperative rehabilitation based on these predictors can effectively reduce adverse outcome risks, offering a solid basis for improving the short- and long-term prognosis of TBAD patients after TEVAR.

## Data Availability

The original contributions presented in the study are included in the article/Supplementary Material, further inquiries can be directed to the corresponding author.
